# Supplementary material for: Antioxidants Discovery for Differentiation of Monofloral Stingless Bee Honeys Using Ambient Mass Spectrometry and Metabolomics Approaches
Source: Foods. 2023 Jun 18;12(12):2404. doi: 10.3390/foods12122404 (PMC10297175; doi:10.3390/foods12122404)
Supplement: Supplementary file 1 [file foods-12-02404-s001.zip › foods-2236682-supplementary.pdf]

Supplementary Table S1

Putatively identified metabolites of stingless bee honeys with reversed phase and HILIC separation in positive and negative ionization modes.

Putative compounds	Separation	Ionization	Rt (min)	m/z	Error (ppm)	Score	Formula	Fragment	Honey
**Alkaloids**									
4-Hydroxyindole	R	Pos	1.10	134.0598	-1.5	35.6	C_8H_7NO	117.0579, 107.0490, 77.0386	All
Crotanecine	R	Pos	1.10	194.0806	3.4	43.0	$C_8H_{13}NO_3$	138.0550136.0760, 126.0550, 114.0550, 112.0398, 84.0444	All
Coronaridine	N	Pos	1.15	339.2086	5.5	36.4	$C_{21}H_{26}N_2O_2$	144.0810, 130.0650, 103.0546	Acacia, Coconut
Indoleacetaldehyde	N	Pos	2.97	160.0762	3.3	44.6	$C_{10}H_9NO$	142.0650, 130.0650, 115.0540, 105.0704, 103.0547, 79.0547	Agarwood, Starfruit
Plantagonine	N	Pos	2.99	178.0868	2.9	43.3	$C_{10}H_{11}NO_2$	160.0760, 142.0650, 132.0810, 130.0650, 115.0540, 105.0700, 103.0540, 91.0542	Acacia, Agarwood, Starfruit
4-Phenylpyridine	N	Pos	3.04	156.0804	-2.2	41.7	$C_{11}H_9N$	115.0535, 106.0660, 106.0701, 103.0546, 91.0546, 79.0544	All
Cinchoninone	N	Pos	3.18	293.1657	2.9	39.0	$C_{19}H_{20}N_2O$	132.0808, 130.0650, 115.0543	Starfruit
8-Isoquinoline methanamine	N	Pos	3.20	158.0962	-1.4	41.6	$C_{11}H_{11}N$	130.0648	All
Swainsonine	N	Pos	3.32	174.1122	-1.4	41.3	$C_8H_{15}NO_3$	156.0995, 120.0809, 118.0666, 103.0548, 91.0545, 79.0547, 77.0390, 69.0334	Coconut, Starfruit
Amabiline/Supinine	N	Pos	3.38	284.1863	2.2	39.6	$C_{15}H_{25}NO_4$	120.0811, 70.0651	Starfruit
1-Acetylindole	N	Pos	3.85	160.0759	1.1	43.3	$C_{10}H_9NO$	145.0509, 118.0651	MC, Agarwood, Starfruit
Salsolidine	R	Pos	4.17	208.1336	1.9	38.4	$C_{12}H_{17}NO_2$	105.0700, 103.0540	MC, Rubber, Agarwood, Starfruit
Indoleacrylic acid	R	Pos	5.02	188.071	1.9	49.2	$C_{11}H_9NO_2$	170.0601, 142.0649, 141.0564, 140.0505, 127.0537, 115.0540, 89.0390	All
6-Methylquinoline	R	Pos	5.02	144.0808	0.5	38.9	$C_{10}H_9N$	118.0655, 115.0546, 91.0546, 89.0390, 77.0390	All
Isoquinoline	R	Pos	5.02	130.0656	3.6	40.4	C_9H_7N	103.0547, 77.0391	All except Acacia, Agarwood
Indole-3-acetonitrile	R	Pos	5.03	157.0774	9.0	41.1	$C_{10}H_8N_2$	146.0604, 130.0651, 117.0572	Coconut
2-Indolecarboxylic acid	R	Pos	5.41	162.0553	1.9	48.1	$C_9H_7NO_2$	144.0440	DMP, Rubber, Coconut, Starfruit
Kynurenic acid	R	Pos	5.41	190.0502	2.0	50.8	$C_{10}H_7NO_3$	162.0554, 144.0448, 116.0500	All
2-Pyrrolidineacetic acid	N	Pos	5.79	130.0864	1.3	42.7	$C_6H_{11}NO_2$	112.0760, 94.0651, 82.0651	Coconut, Starfruit
Harmane	R	Pos	6.56	183.0911	-3.0	39.1	$C_{12}H_{10}N_2$	168.0700, 167.0600, 140.0500	All
Perlolyrine	R	Pos	9.20	265.0988	6.3	46.0	$C_{16}H_{12}N_2O_2$	247.0870, 205.0763	All
Mukonidine	R	Pos	9.57	242.0802	-3.9	36.3	$C_{14}H_{11}NO_3$	130.0655, 128.0490	Agarwood
2,2'-Bipyridylamine	R	Pos	9.57	172.0872	1.5	47.0	$C_{10}H_9N_3$	156.0562, 145.0764, 143.0605	Acacia, Agarwood
Palmatine	R	Pos	11.24	353.1613	-2.4	38.0	$C_{21}H_{22}NO_4+$	322.1440	All
Flazine	R	Pos	11.71	309.0883	4.4	38.1	$C_{17}H_{12}N_2O_4$	263.0820	All except Agarwood
Strictosamide	R	Pos	11.80	499.2091	3.1	41.2	$C_{26}H_{30}N_2O_8$	337.1550, 267.1130	All except Agarwood
3-Methyldioxyindole	R	Pos	11.93	164.0719	8.1	42.8	$C_9H_9NO_2$	138.0556, 136.0762, 121.0654	All
Melochinone	R	Pos	12.54	332.1651	1.8	32.0	$C_{22}H_{21}NO_2$	232.1124	All
**Flavonoids**									
Aliarin	N	Pos	1.09	417.1549	1.3	38.5	$C_{22}H_{24}O_8$	355.1180, 121.0650, 105.0330	All
4',5-Dihydroxy-7-methoxyflavanone	N	Neg	1.77	285.0770	0.6	34.7	$C_{16}H_{14}O_5$	119.0505	All
Quercitrin	N	Neg	3.03	447.0901	-7.2	41.9	$C_{21}H_{20}O_{11}$	301.0349, 271.0232	All
Saponarin	N	Neg	3.81	593.1510	-0.3	42.2	$C_{27}H_{30}O_{15}$	431.0993	All
Linarin	N	Neg	3.83	591.1715	-0.8	36.8	$C_{28}H_{32}O_{14}$	151.0038, 107.0141	MC
Vitexin-2"-O-rhamnoside	N	Pos	4.14	579.1716	1.3	36.8	$C_{27}H_{30}O_{14}$	139.0540, 115.0544, 109.0289, 97.0288, 91.0542, 81.0334, 69.0334	MC, Starfruit
Chrysanthemin	R	Pos	7.19	449.1086	1.7	40.6	$C_{21}H_{20}O_{11}$	287.0558	Coconut
3,6,2',4'-Tetrahydroxyflavone	R	Pos	7.19	287.0567	5.8	38.5	$C_{15}H_{10}O_6$	115.0545, 91.0543	Rubber, Coconut
Kaempferol 3-glucosyl-(1->4)-rhamnosyl-(1->2)-glucoside	R	Pos	7.19	757.2195	1.2	41.5	$C_{33}H_{40}O_{20}$	449.1080, 287.0550	Coconut

Cyanin	R	Pos	7.81	611.1617	1.6	39.9	$C_{27}H_{30}O_{16}$	287.0558	Coconut
6-Methoxyluteolin	R	Pos	7.93	317.0660	1.4	50.6	$C_{16}H_{12}O_{7}$	302.0428, 273.0399	Acacia, Coconut, Starfruit
Isorhamnetin 3-galactosyl-(1->4)-rhamnosyl-(1->6)-galactoside	R	Pos	7.93	787.2300	1.1	40.1	$C_{34}H_{42}O_{21}$	317.0660	Rubber, Acacia, Coconut, Starfruit
Tamarixetin 3-glucosyl-(1->2)-galactoside	R	Pos	7.93	641.1722	1.5	38.8	$C_{28}H_{32}O_{17}$	317.0660	Acacia, Coconut
Rutin	R	Pos	8.19	611.1617	1.7	40.4	$C_{27}H_{30}O_{16}$	303.0533, 287.0557	All
Quercetin 3-isorhamninoside	R	Pos	8.19	757.2192	0.9	38.9	$C_{33}H_{40}O_{20}$	303.0500	Acacia, Coconut, Starfruit
Kaempferol-7-O-neohesperidoside	R	Neg	8.35	593.1510	-0.4	40.6	$C_{27}H_{30}O_{15}$	284.0331	All
3,5,7,3',4'-Pentahydroxyflavanone	R	Neg	8.83	303.0505	-1.9	39.3	$C_{15}H_{12}O_{7}$	150.0327, 125.0248	All except MC
Kaempferol 3-O-rutinoside	R	Pos	8.92	595.1666	1.4	40.3	$C_{27}H_{30}O_{15}$	287.0560	Coconut
Homoeriodictyol	R	Neg	8.99	301.0711	-2.2	38.9	$C_{16}H_{14}O_{6}$	147.0404	Coconut
Luteolin-4'-O-glucoside	R	Neg	9.37	447.0926	-1.6	39.5	$C_{21}H_{20}O_{11}$	300.0271, 271.0241, 255.0290, 245.0434, 211.0410, 178.9978	All
5,7,3',4',5'-Pentahydroxyflavone	R	Pos	9.38	303.0506	2.3	44.6	$C_{15}H_{10}O_{7}$	285.0399, 257.0450, 229.0500, 153.0184, 115.0550, 104.9588, 91.0548	All except Acacia
Genistin	R	Neg	10.25	431.0975	-1.9	39.8	$C_{21}H_{20}O_{10}$	268.9000	All except Acacia, Coconut
Sphenostylin A	R	Pos	11.57	399.1803	0.1	40.2	$C_{23}H_{26}O_{6}$	293.1360	All
7,3',4',5'-Tetrahydroxyflavone	R	Neg	11.65	285.0399	-1.8	36.6	$C_{15}H_{10}O_{6}$	148.9000	All
Isomucronulator 7-O-glucoside	R	Pos	11.97	465.1760	1.0	37.6	$C_{23}H_{28}O_{10}$	271.0960	All
Oaxacacin	R	Pos	16.47	337.1452	5.2	34.9	$C_{21}H_{20}O_{4}$	115.0540, 105.0700, 77.0386	All
Mangostine	R	Neg	17.60	409.1651	-1.3	38.9	$C_{24}H_{26}O_{6}$	325.0715	All except Starfruit
Garcinol	R	Pos	17.62	603.3689	1.5	42.2	$C_{38}H_{50}O_{6}$	301.1429, 287.0534	DMP, Rubber, Acacia, Coconut
**Phenolic acids**									
2-Methylhippuric acid	N	Neg	2.43	192.0663	-1.4	40.0	$C_{10}H_{11}NO_{3}$	148.0759, 91.0553	All
2-Methylbenzoic acid	R	Neg	3.49	135.0446	-3.8	39.6	$C_{8}H_{8}O_{2}$	119.0489, 91.0547	Rubber, Acacia
trans-Caffeic acid [apiosyl-(1->6)-glucosyl] ester	R	Pos	3.62	475.1426	-4.2	38.3	$C_{20}H_{26}O_{13}$	365.1080, 109.0280, 107.0490	All except Agarwood
3'-Methoxyfukiic acid	R	Pos	3.88	287.0744	-6.0	36.6	$C_{12}H_{14}O_{8}$	163.0390	All except Agarwood, Coconut
3-Caffeoyl-1,5-quinolactone	R	Pos	4.88	337.0902	-4.8	38.9	$C_{16}H_{16}O_{8}$	163.0390, 135.0440	MC, Rubber, Acacia
4-p-Coumaroylquinic acid	N	Pos	4.89	339.1072	-0.6	39.8	$C_{16}H_{18}O_{8}$	245.0636, 145.0499	Acacia, Coconut, Starfruit
Salicylic acid	R	Neg	5.78	137.0239	-3.6	42.6	$C_{7}H_{6}O_{3}$	93.0348	All
DL-Mandelic acid	R	Neg	5.78	151.0395	-3.6	41.2	$C_{8}H_{8}O_{3}$	135.0449, 107.0499, 93.0340	All except Coconut
5-Acetyl-2-hydroxybenzoic acid	R	Neg	6.53	179.0354	2.5	41.9	$C_{9}H_{8}O_{4}$	135.0456	Rubber, Coconut, Agarwood
trans-Cinnamic acid	R	Pos	6.55	149.0602	3.4	38.7	$C_{9}H_{8}O_{2}$	103.0539, 77.0385	Starfruit
2-Hydroxy-4-methylbenzoic acid	R	Neg	7.41	151.0395	-3.5	65.0	$C_{8}H_{8}O_{3}$	108.0531, 107.0497	All except Coconut
N-Feruloyltyramine	R	Pos	8.63	314.1394	2.2	43.0	$C_{18}H_{19}NO_{4}$	135.0446, 107.0490, 105.0330, 79.0542, 77.0390, 51.0229	Agarwood
Cinnamyl butyrate	R	Pos	8.77	205.1225	0.7	39.8	$C_{13}H_{16}O_{2}$	115.0543, 106.9924, 105.0703, 93.0703, 77.0391	Rubber, Acacia, Agarwood, Starfruit
Cinnamyl anthranilate	R	Pos	10.81	254.1180	1.8	36.1	$C_{16}H_{15}NO_{2}$	91.0542	All except Coconut, Starfruit
**Other polyphenols**									
2,4,5-Trihydroxytoluene	R	Pos	1.09	141.0541	-3.8	38.2	$C_{7}H_{8}O_{3}$	69.0335	All except Agarwood, MC
2-Phenylpropionaldehyde	N	Pos	2.39	135.0805	0.1	41.1	$C_{9}H_{10}O$	115.0543, 107.0504, 105.0702, 79.0546, 77.0389	All
2,6-Dihydroxy-4-methoxytoluene	N	Pos	3.84	155.0709	4.1	40.2	$C_{8}H_{10}O_{3}$	99.0452, 85.0282, 79.0547, 69.0339	All except Agarwood, DMP
1,4-Diethylbenzene	R	Pos	4.39	135.1174	4.3	39.5	$C_{10}H_{14}$	105.0703	Agarwood
Miraxanthin-III	R	Pos	6.27	331.1296	2.2	36.0	$C_{17}H_{18}N_{2}O_{5}$	103.0540, 91.0542, 77.0386	Coconut
4-Hydroxycoumarin	R	Pos	6.54	163.0394	2.4	38.3	$C_{9}H_{6}O_{3}$	84.0962	Agarwood
Salidroside	R	Pos	6.82	323.1109	2.4	38.7	$C_{14}H_{20}O_{7}$	181.0861, 121.0647	Agarwood
2',3',4'-Trihydroxyacetophenone	N	Neg	6.94	167.0346	-2.4	39.5	$C_{8}H_{8}O_{4}$	125.0244, 97.0284	All

3-(4-Methylphenyl)-2-propenal	R	Pos	7.65	147.0809	2.9	38.8	$C_{10}H_{10}O$	129.0700	Rubber
2-Butyl-3-phenyl-2-propen-1-al	R	Pos	7.66	189.1277	1.5	38.8	$C_{13}H_{16}O$	91.0542	Acacia
4-Methoxybenzyl acetate	R	Pos	7.70	181.0858	-0.5	47.5	$C_{10}H_{12}O_3$	139.0750, 137.0600, 121.0650, 109.0650, 107.0490	All
(Dimethoxymethyl)benzene	R	Pos	7.70	153.0914	2.8	40.4	$C_9H_{12}O_2$	137.0600, 123.0800, 121.065	DMP, Rubber
4-Phenyl-2-butenal	R	Pos	8.76	147.0808	2.6	40.2	$C_{10}H_{10}O$	129.0700, 115.0540, 105.0700, 93.0699, 91.0542, 79.0542, 77.0386	DMP, Rubber, Agarwood
(2-Methoxyethyl)benzene	R	Pos	9.36	137.0966	3.4	38.4	$C_9H_{12}O$	105.0700, 91.0542, 77.0386	Agarwood
Benzyl butyl ether	R	Pos	11.25	165.1275	0.8	38.5	$C_{11}H_{16}O$	91.0542	MC
Matairesinol	R	Pos	12.57	359.1495	1.7	41.2	$C_{20}H_{22}O_6$	237.1120, 163.0750, 137.0600, 107.0490, 91.0542, 77.0386	DMP, Acacia, Coconut
**Terpenoids**									
Dihydrocarvone	N	Pos	1.76	153.1278	2.5	43.9	$C_{10}H_{16}O$	135.1172, 115.0545, 105.0703, 103.0549, 95.0855, 91.0545, 81.0698, 79.0545, 77.0388, 67.0545	Rubber, Acacia, Coconut, Starfruit
Carvacrol	N	Pos	1.89	151.1115	-1.5	46.8	$C_{10}H_{14}O$	135.0800, 133.1010, 123.0800, 121.1010, 119.0860, 109.0650, 107.0490, 105.0700, 95.0855, 93.0699, 91.0542, 81.0699, 79.0542, 77.0386	All except Coconut
Oleoside dimethyl ester	N	Pos	2.86	419.1566	4.2		$C_{18}H_{26}O_{11}$	257.1020	All
Neryl acetate	R	Pos	3.47	219.1345	-5.5	36.5	$C_{12}H_{20}O_2$	79.0551	Coconut
Harpagide	R	Pos	4.03	387.1272	2.9	36.5	$C_{15}H_{24}O_{10}$	203.0537, 97.0289	Rubber, Acacia
Foeniculoside VII	R	Pos	5.23	349.1863	1.6	36.8	$C_{16}H_{28}O_8$	227.1280	Rubber, Acacia, Starfruit
p-Mentha-1,3,8-triene	R	Pos	6.23	135.1172	3.0	39.2	$C_{10}H_{14}$	107.0861, 93.0707, 77.0394	All except Coconut, DMP
Picrotoxinin	R	Pos	6.28	293.1009	-3.5	37.8	$C_{15}H_{16}O_6$	-	All except Acacia
6-Hydroxy-2-bornanone glucoside	R	Pos	7.34	353.1572	0.4	39.6	$C_{16}H_{26}O_7$	169.1220	MC, Rubber, Agarwood, Starfruit
(+)-Iridodial	R	Pos	8.44	169.1227	2.4	44.5	$C_{10}H_{16}O_2$	151.1120, 139.1120, 123.1170, 121.1010, 109.1010, 107.0860, 105.0700, 95.0855, 93.0699, 81.0699, 79.0542	Agarwood
Thujone	R	Pos	8.77	153.1276	1.7	45.6	$C_{10}H_{16}O$	135.1173, 107.0854, 105.0708, 93.0700, 91.0548, 81.0702, 79.0547, 77.0390	DMP, Rubber, Acacia, Starfruit
xi-3-(4-Isopropylphenyl)-2-methylpropanal	R	Pos	8.95	191.1434	1.9	38.3	$C_{13}H_{18}O$	133.1010, 115.0540	Agarwood
p-Mentha-1,3,5,8-tetraene	R	Pos	8.96	133.1016	3.2	38.4	$C_{10}H_{12}$	115.0546	Agarwood
(+-)-2-cis-4-trans-Abscisic acid	R	Neg	11.25	263.1282	-2.5		$C_{15}H_{20}O_4$	219.1393, 204.1159, 201.1288, 153.0925, 152.0846	All
Achillin	R	Pos	11.26	247.1334	2.1	38.5	$C_{15}H_{18}O_3$	128.0613, 115.0542, 105.0705, 91.0549, 77.0395	DMP, Rubber, Acacia, Agarwood
8-Isobutanoylneosolaniol	R	Pos	12.85	453.2093	-5.7	26.7	$C_{23}H_{32}O_9$	271.0810	All
Asiatic acid	R	Neg	16.19	487.3414	-3.1	38.3	$C_{30}H_{48}O_5$	455.3163, 393.3163	All
**Vitamins**									
Lumichrome	N	Neg	2.13	241.0729	-0.9	46.3	$C_{12}H_{10}N_4O_2$	198.0676	All
(-)-Riboflavin	R	Pos	6.70	377.1463	2.1	40.4	$C_{17}H_{20}N_4O_6$	243.0900	Rubber, Acacia, Agarwood, Starfruit
Cholacalcioic acid	R	Pos	18.87	373.2738	0.2	39.5	$C_{24}H_{36}O_3$	259.1690	DMP, Rubber

Rt: Retention time; R: Reversed phase; N: Normal phase; Pos: Positive; Neg: Negative; MC: Mexican creeper; DMP: Dwarf mountain pine

Supplementary Table S2

The potential discriminant metabolites for stingless bee honeys obtained from OPLS-DA.

Honey	Putative compounds	p[1]	p(corr)[1]	VIP	LogFC vs Others
Acacia	2-Butyl-3-phenyl-2-propen-1-al	-2.02	-0.98	1.8	5.15
	Tamarixetin 3-glucosyl-(1->2)-galactoside	-2.22	-0.76	2.0	4.38
	2-Indolecarboxylic acid	2.20	0.67	2.0	-6.41
	6-Methoxyluteolin	-2.51	-0.59	2.2	3.34
	Isorhamnetin 3-galactosyl-(1->4)-rhamnosyl-(1->6)-galactoside	-2.63	-0.58	2.4	3.47
	Matairesinol	-2.93	-0.66	2.6	4.37
Agarwood	4-Hydroxycoumarin	-3.01	-0.76	1.5	4.21
	1-Acetylindole	-3.27	-0.55	1.6	2.73
	Plantagonine	-3.36	-0.55	1.7	3.34
	N-Feruloyltyramine	-3.65	-0.72	1.8	5.03
	Indoleacetaldehyde	-3.84	-0.65	1.9	3.72
	3'-Methoxyfukiic acid	4.03	0.65	2.0	-7.34
	2,2'-Bipyridylamine	-3.99	-0.83	2.0	6.13
	(+)-Iridodial	-4.04	-0.91	2.0	6.72
	Garcinol	4.57	0.52	2.2	-11.86
	Flazine	5.19	0.96	2.6	-9.66
	2-Pyrrolidineacetic acid	7.42	0.67	3.8	-18.31
Coconut	3,6,2',4'-Tetrahydroxyflavone	-3.24	-0.61	1.6	4.62
	Neryl acetate	-3.28	-0.95	1.6	5.85
	Cyanin	-3.78	-0.96	1.8	6.74
	2-Hydroxy-4-methylbenzoic acid	4.26	0.94	2.0	-7.68
	Kaempferol 3-O-rutinoside	-4.28	-0.94	2.1	7.41
	Quercetin 3-isorhamninoside	-4.52	-0.76	2.2	6.35
	Kaempferol 3-glucosyl-(1->4)-rhamnosyl-(1->2)-glucoside	-5.16	-0.97	2.5	9.04
DMP	(-)-Riboflavin	1.50	0.55	1.7	-5.69
	4-Phenyl-2-butenal	-1.57	-0.64	1.7	2.77
	Oleoside dimethyl ester	-1.85	-0.67	2.0	3.31
	6-Hydroxy-2-bornanone glucoside	1.79	0.55	2.0	-5.61
	5,7,3',4',5'-Pentahydroxyflavone	-2.00	-0.77	2.2	4.97
	Genistin	-2.08	-0.72	2.3	4.86
	(Dimethoxymethyl)benzene	-2.13	-0.69	2.3	3.14
	Luteolin-4'-O-glucoside	-2.30	-0.75	2.5	5.26
	Cholacalcioic acid	-2.72	-0.52	2.9	3.29
MC	3,5,7,3',4'-Pentahydroxyflavanone	1.99	0.72	1.7	-6.03
	Cinnamyl butyrate	4.24	0.54	3.3	-14.41
	Linarin	-5.49	-0.76	4.8	8.14
Rubber	2-Methylbenzoic acid	1.63	0.57	1.5	2.85
	Strictosamide	1.79	0.63	1.7	2.97
	DL-Mandelic acid	1.87	0.65	1.8	3.61
	Achillin	2.01	0.59	1.9	3.60
	3-(4-Methylphenyl)-2-propenal	2.25	0.89	2.1	5.01
	3-Caffeoyl-1,5-quinolactone	2.74	0.72	2.6	5.22
Starfruit	trans-Cinnamic acid	3.13	0.99	2.3	8.23
	2-Phenylpropionaldehyde	-0.55	-0.67	2.7	-1.57
	Mangostine	-4.70	-0.93	3.6	-13.03
	Amabiline/Supinine	5.08	0.70	4.0	9.00
	Cinchoninone	6.11	0.77	4.5	8.80

MC: Mexican creeper; DMP: Dwarf mountain pine; VIP: Variables importance in the projection
